# The Relationship between the Expression of GATA4 and GATA6 with the Clinical Characteristics and Prognosis of Resectable Pancreatic Adenocarcinoma

**DOI:** 10.3390/biomedicines11020252

**Published:** 2023-01-18

**Authors:** Victoria Heredia-Soto, Laura Gutiérrez-Sainz, Ismael Ghanem, Laura Guerra, Elena Palacios, Marta de Uribe, Lucía Trilla-Fuertes, María de Miguel, Paloma Cejas, Laura Medina, José Miguel Calderón, David Viñal, Marta Mendiola, Jaime Feliu

**Affiliations:** 1 Translational Oncology Research Laboratory, Biomedical Research Institute, La Paz University Hospital, IdiPAZ, Paseo de la Castellana 261, 28046 Madrid, Spain; 2 Oncology Department, La Paz University Hospital, IdiPAZ, Paseo de la Castellana 261, 28046 Madrid, Spain; 3 Pathology Department, La Paz University Hospital, Paseo de la Castellana 261, 28046 Madrid, Spain; 4 Molecular Oncology Lab, Instituto de Genética Médica y Molecular-INGEMM, IdiPAZ, La Paz University Hospital, 28046 Madrid, Spain; 5 Center for Functional Cancer Epigenetics, Dana-Farber Cancer Institute, Boston, MA 02215, USA; 6 UGCI Medical Oncology Hospital Virgen de la Victoria, Instituto de Investigación Biomédica de Málaga, 29010 Málaga, Spain; 7 Pathology Department, Fuenlabrada University Hospital, 28942 Madrid, Spain

**Keywords:** pancreatic ductal adenocarcinoma, GATA6, GATA4, prognostic, survival, carcinogenesis

## Abstract

GATA4 and GATA6 are transcription factors involved in the differentiation and development of PDAC. GATA6 expression is related to the classic molecular subtype, while its absence is related to the basal-like molecular subtype. The aim was to determine the clinical utility of IHC determination of GATA4 and GATA6 in a series of patients with resected PDAC. GATA4 and GATA6 expression was studied by IHC in TMA samples of normal tissue, PanIN, tumor tissue and lymph node metastases from a series of 89 patients with resected PDAC. Its relationship with clinicopathologic variables and the outcome was investigated. Seventy-two (81%) tumors were GATA6+ and 37 (42%) were GATA4+. While GATA4 expression was reduced during tumor progression, GATA6 expression remained highly conserved, except in lymph node metastases. All patients with early stages and well-differentiated tumors were GATA6+. The absence of GATA4 expression was related to smoking. Patients with GATA4+ or GATA6+ tumors had significantly lower Ca 19.9 levels. The expression of GATA4 and GATA6 was related to DFS, being more favorable in the GATA4+/GATA6+ group. The determination of the expression of GATA4 and GATA6 by IHC is feasible and provides complementary clinical and prognostic information that can help improve the stratification of patients with PDAC.

## 1. Introduction

Pancreatic ductal adenocarcinoma (PDAC) is an aggressive disease with a dismal prognosis. The absence of early diagnostic markers and successful therapeutic options is related to the poor prognosis of this disease. Although significant efforts have been made in recent decades to develop new treatments, no substantial advances have been made and 5 year survival remains less than 10% [1].

The only current PDAC curative treatment is an adequate surgery that, unfortunately, is only performed in a limited subgroup of patients with localized disease. However, even after surgery, 80% of the patients will relapse [2]. Adjuvant chemotherapy can increase disease free survival (DFS) and overall survival (OS) but, even so, 60% of patients relapse within three years after surgery [3]. Nowadays there are no established criteria to select chemotherapy beyond the performance status.

Classifications based on clinicopathological features fail to improve survival and other stratification systems have been proposed based on molecular characteristics. Recent studies have demonstrated that PDAC is a much more heterogeneous tumor than expected from the molecular point of view [4,5,6]. Different proposals have been developed, distinguishing between two to five molecular subgroups, with two of them in common among the proposed classifications: the so-called “classical” and “basal-like”. The first one is composed of tumors with well to moderately differentiated cells that retain the expression of pancreatic endoderm markers, where they are originated, express transcription factors as GATA6, and overexpress genes related to cellular adhesion. The second subtype, “basal-like” or alternatively named “quasi-mesenchymal” or “squamous”, is composed of poorly differentiated cells with an absence of pancreatic tissue markers expression. These tumors are characterized by the activation of aberrant differentiation pathways, as keratin production, the formation of a stratified epithelium, and epithelial to mesenchymal transition (EMT) [5]. Although other molecular subtypes have been proposed, they probably reflect the presence of non-tumor cells, such as inflammatory cells and cells of the endocrine pancreas [5,7,8].

In localized disease, the classical subtype predominates, although the reported frequencies vary widely among the series considered, ranging between 46% and 90% [8,9]. Some authors have found a relationship between subtypes and survival, with the classical subtype related to a better outcome and the basal-like to a worse survival [5]. The identification of molecular subtypes would be interesting for prognosis but also to establish more successful therapeutic approaches. In this regard, it has been suggested that patients within classical subtype would respond better to 5-fluorouraracil (5FU) based schemes, while the basal-like will respond better to gemcitabine containing options [8].

Large prospective clinical trials would be necessary to validate these observations. Additionally, most of the proposed molecular classifications are based on transcriptomic data that make their implantation difficult in the clinical routine. The identification of surrogate markers with reproducible data and which are easy to perform constitute a challenge. In this regard, an immunohistochemical approach (IHC) is an option, when reproducible data are obtained. GATA6 is a transcription factor that maintains the cellular differentiation of the pancreatic epithelium, and its overexpression has been related to the classical subtype [10]. Therefore, the identification of GATA6 is considered a surrogate marker of the classic subtype and can be carried out in a simple way using in situ hybridization and immunohistochemical techniques [10,11]. Another transcription factor involved in pancreatic development and PDAC progression is GATA4 [12,13]. Actually, during early mouse development, GATA4 and GATA6 are expressed in the extraembryonic endodermal lineages including the visceral and parietal endoderm [14]. Both transcription factors’ expression seems to be related, since GATA6 is an upstream regulator of GATA4, and its deletion results in the absence of GATA4 expression, while GATA4 deletion promotes an upregulation of GATA6 expression [15].

The aim of this study is to investigate the clinical utility of IHC determination of GATA4 and GATA6 transcription factors in a series of patients with resected PDAC, as well as to understand the relationship of GATA4 and GATA6 with PDAC progression.

## 2. Materials and Methods

### 2.1. Patient Samples and Clinical Data

A retrospective cohort of patients diagnosed with localized PDAC (stage I-II according to the 7th edition of TNM) at La Paz University Hospital and Fuenlabrada University Hospital between 2010 and 2018 were identified as eligible for the study. Other inclusion criteria were age ≥ 18, having undergone surgery with curative intent, having received adjuvant treatment with a gemcitabine-based regimen, and the availability of FFPE primary tumor of adequate quality for study. Patients who died within 30 days of surgery were excluded.

Within two months after surgery, patients started adjuvant treatment with gemcitabine +/− capecitabine for 6 months. Subsequently, they were followed up for 5 years by CT and analysis (including Ca 19.9) every 3 months during the first two years and then every 6 months until completing 5 years.

Demographic variables (age, sex), predisposing factors (history of alcohol and tobacco consumption, diagnosis of diabetes, and body mass index (BMI)), variables related to the tumor (location, pathological stage, degree of differentiation, and presence of lymphovascular and perineural infiltration), type of resection, ECOG PS, analytical variables (Ca 19.9 and neutrophil/lymphocyte ratio (INL)), adjuvant treatment received, DFS, location of relapse, and OS were all recorded.

The study was approved by the Ethical Committees of La Paz University Hospital (IRB number: 1349) and Fuenlabrada University Hospital (IRB number: APR17/62).

### 2.2. Immunohistochemistry

Optimal tissue blocks were selected by an expert pathologist on haematoxylin and eosin (H&E) slides. For each patient, representative areas of normal tissue, tumor tissue, preneoplastic lesions grade 2–3 (PanIN), and lymph node metastases were selected and included in tissue microarrays (TMA). For each sample, two representative cores of 1.2 mm in diameter were arrayed into a receptor block using a TMA workstation (Beecher Instruments, Silver Spring, MD, USA) as previously described [16]. For IHC assays, 4 µm sections of the TMAs were used. Slides were deparaffinized and rehydrated. Antigen retrieval was performed in a DAKO PT Link, incubated with specific primary antibodies: GATA-4 clone 532020 ref. n° MAB2606 and GATA-6 ref. n° AF1700 (R&D Systems, MN, USA), detected with a Dako Envision Plus kit, and counterstained with haematoxylin. All reagents are from Dako (Agilent, CA, USA). The staining was assessed by two independent pathologists, blinded to the origin of the samples. Both the intensity of the staining (0—negative, 1—weak, and 2—strong) and the percentage of positive cells of each tissue core were determined. An H-score (intensity x% positive cells) was calculated, ranging from 0 to 300). H-scores of <30 were considered negative [17].

### 2.3. Statistical Analyses

Demographic and baseline characteristics were listed and summarized using descriptive statistics. All data are presented as mean ± SD. Differences between groups were evaluated with independent *t* tests for continuous variables and χ^2^ tests for categorical variables. Yates’ correction was applied when necessary. DFS and OS curves were plotted using the Kaplan–Meier method and HRs were calculated using Cox proportional hazard regression, with P values calculated using the Wald statistics. We compared the Kaplan–Meier survival curves between groups using a log-rank test. Multivariable Cox regression was used to estimate the prognostic factors and their weights, and Cox regression was also used for univariate analyses on continuous and categorical prognostic factors. The proportional hazards assumption was verified by test of correlations with time and examination of residual plots. All statistical tests were two-sided, and P values less than 0.05 were considered to be statistically significant. Analyses were carried out with SPSS software (version 18; SPSS, Chicago, IL, USA).

## 3. Results

### 3.1. Patients’ Characteristics

A total of 122 patients with resectable PDAC underwent surgery between 2010 and 2018. From these, 7 patients’ follow-up was lost early after surgery, 4 patients had metastases detected during surgery, 6 died in the first two months post-surgery, 10 did not receive adjuvant treatment, and 6 did not have an adequate tumor sample for IHC studies, and all these patients were excluded from the analysis; thus, the study finally included 89 patients.

Baseline patient characteristics are summarized in Table 1. The median age was 64 years (range: 29–84). Forty-six (52%) patients were women. Forty-one (46%) patients had a smoking history and 28 (33%) had a history of alcohol use. Twenty (23%) patients had a history of diabetes and 32 (36%) showed signs of chronic pancreatitis in histology. Most of the patients had stage IIB (76%), and they had a moderately differentiated tumor (81%). In 50 (56%) patients, surgery was R1. Seventy-two (81%) patients had GATA6-positive tumors, and 37 (42%) had GATA4-positive tumors. There were 19 (21%) GATA6+/GATA4+, 8 (9%) GATA6−GATA4−, 55 (62%) GATA6+/GATA4−, and 7 (8%) GATA6−/GATA4+ tumors.

### 3.2. Expression of GATA4 and GATA6 in Normal Tissue, PanIN, Tumor Tissue and Lymph Node Metastasis

**Figure 1:**
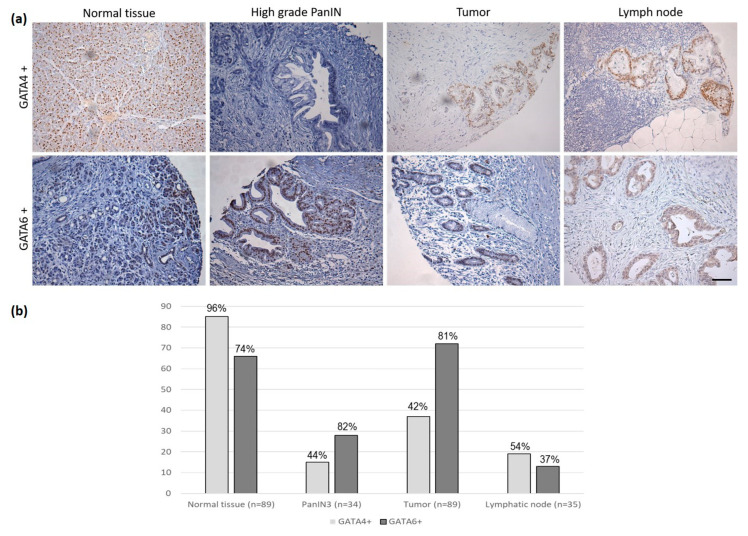
GATA4 and GATA6 expression in normal tissue, PanIN, tumor tissue and lymph node metastasis: (**a**) GATA4+ (upper panel) and GATA6+ (lower panel) immunohistochemistry images. Scale bar represents 100 µm; (**b**) Graphical representation of GATA4+ and GATA6+ distribution.

In order to study the changes in the expression of GATA4 and GATA6 during the process of tumor development, their expression was studied in healthy tissue, PanIN, tumor tissue, and metastatic lymph node. We had 89 samples of tumor tissue paired with 89 samples of healthy peritumoral tissue, 34 of PanIN grade 2–3, and 35 of lymph node metastasis. We observed that while 85/89 (96%) healthy tissue samples expressed GATA4, this occurred with GATA6 in 66/89 (74%) (*p* < 0.001). Regarding PanIN, 15/34 (44%) samples expressed GATA4, while this occurred in 28/34 (82%) with GATA6 (*p* < 0.001). Regarding lymph node metastases, 19/35 (54%) expressed GATA4, while 13/35 (37%) expressed GATA6 (*p* = NS) (Figure 1a,b).

When the differences in the expression of GATA4 and GATA6 between the paired samples of normal tissue and PanIN were analyzed, we observed that in 15/34 (44%) samples the expression of GATA4 was lost, while that of GATA6 was preserved in the 34 cases analyzed (*p* < 0.001). When the differences of GATA4 and GATA6 were studied in the paired samples of PanIN and tumor tissue, it was observed that the expression of GATA4 was lost in 5 (15%) cases, remained the same in 25 (73%) and increased in 4 (12%). Regarding the expression of GATA6, it was lost in 4 (12%), unchanged in 26 (76%), and increased in 4 (12%) (*p* = NS).

Finally, regarding the differences between tumor tissue and lymph node metastasis, it was observed that the expression of GATA4 was lost in 4 (12%) tumors, remained the same in 26 (74%) and became positive in 5 (14%). Regarding the expression of GATA6, in 14 (40%) tumors it becomes negative, in 20 (57%) it remains unchanged and in one case (3%) it becomes positive (*p* = 0.01).

### 3.3. Characteristics of the Tumors According to the Expression of GATA4 and GATA6

Table 2 shows the clinical characteristics of the patients based on the tumor expression of GATA6 and GATA4. No differences were found in the expression of GATA6 depending on the etiological factor considered. However, we observed that 54% of the tumors developed in non-smokers were GATA4+, while this only occurred in 37% of non-smokers (*p* = 0.03). In relation to the stage, it should be noted that the 8 tumors (100%) in stage IA/IB expressed GATA6, while this occurred in 79% of the tumors with stages IIA/IIB (*p* = NS). Regarding GATA4 expression, 25% of stage IA/IB tumors were positive and 43% of stages IIA/IIB (*p* = NS). No statistically significant relationships were detected between the degree of cell differentiation and the expression of GATA6 and GATA4, although it should be noted that the 8 well-differentiated tumors were GATA6+, while only 3 of them were GATA4+. In addition, while the absence of expression of GATA6 tended to be related to perineural infiltration (25% vs. 7%; *p* = 0.065) the positive expression of GATA4 was the one that was significantly related to the presence of perineural infiltration (58% vs. 10%; *p* = 0.01). On the other hand, patients with GATA6+ tumors showed significantly lower Ca 19.9 levels than patients with GATA6− tumors [606 IU/mL (SD 1010) vs. 1501 IU/mL (SD 2413); *p* = 0.03]. Similarly, patients with GATA4+ tumors had significantly lower Ca 19.9 levels than patients with GATA4- tumors [284 IU/mL (SD 340) vs. 982 IU/mL (SD 1610); *p* = 0.01]. In relation to the INL index, no differences were detected based on the expression of GATA6, but patients with GATA4+ tumors had a significantly higher INL than patients with GATA4− tumors [7.8 (SD 2.5) vs. 3.5 (SD 2.9); *p* = 0.02].

### 3.4. Prognostic Value of GATA4 and GATA6 Expression in Pancreatic Tumor

**Figure 2:**
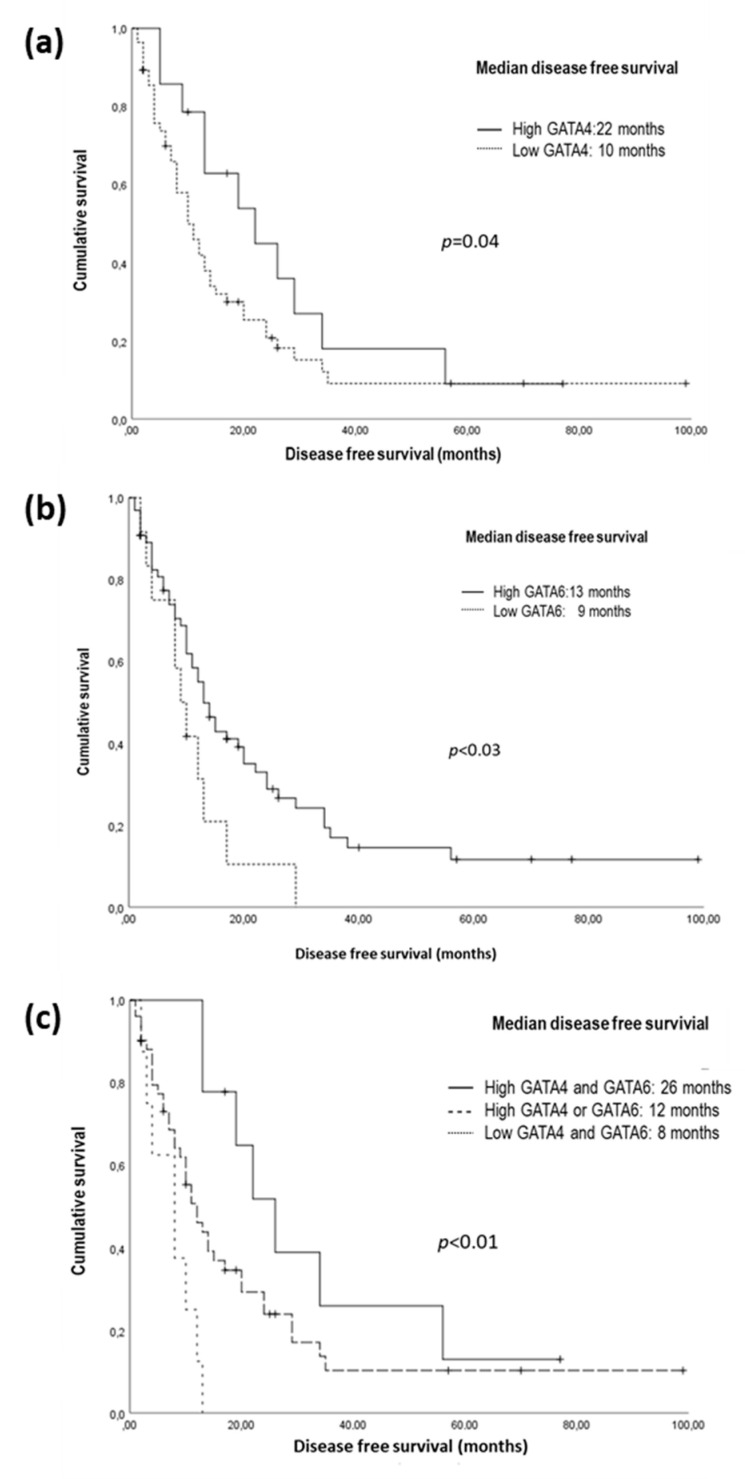
Kaplan–Meier plots of disease-free survival (DFS) by GATA4 and GATA6 tumoral expression. (**a**) Kaplan–Meier plot of DFS by GATA4 expression; (**b**) Kaplan–Meier plot of DFS by GATA6 expression; (**c**) Kaplan–Meier plot of DFS by combined GATA4 and GATA6 expression.

The potential prognostic value of GATA4 and GATA6 expression for both DFS and OS was evaluated. We found that tumor expression of both GATA4 and GATA6 were significantly related to DFS (Figure 2A,B). Furthermore, when the expression of both transcription factors was considered together, patients with GATA4+/GATA6+ tumors had significantly higher median DFS than those expressing only one or neither of them [26 m (95% CI: 16.8–35.1) vs. 12m (95% CI 8.5–12.7) vs. 8m (95% CI 1.8–10.1); *p* < 0.01] (Figure 2C).

Other prognostic factors related to DFS that we identified in the univariate analysis were lymph node invasion, TNM stage, and Ca 19.9 level. In the Cox model, only three independent variables were directly associated with DFS: TNM stage, Ca 19.9 value, and the joint expression of GATA4 and GATA6 (Table 3). When OS was analyzed based on GATA4 and GATA6 expression, we found that patients with simultaneous GATA4 and GATA6 expression tended to have higher OS than those expressing only one or none of the transcription factors respectively [23 m (95% CI: 12.1–33.8) vs. 18 m (95% CI: 13.5–22.5) vs. 12 m (95% CI: 5.2–18.7); *p* = 0.078]. The only variable that showed a significant relationship with OS was the level of Ca19.9 (Table 4).

## 4. Discussion

The survival of patients with PDAC has barely changed in the last 50 years. Therefore, it is necessary to deepen the knowledge of the molecular biology of PDAC and its clinical implications. The results obtained in our study suggest that the tumor expression of the transcription factors GATA4 and GATA6, measured by IHC techniques, is modified throughout tumor development. Furthermore, in resected PDAC, their expression is related to certain clinical and pathological characteristics of the patient and the tumor, respectively, as well as to prognosis.

In our series, 81% of patients had tumors that expressed GATA6, providing similar results to those described by other authors using transcriptomics techniques in localized PDAC [8]. Other authors, however, reported that it was expressed in a slightly lower percentage (62% of patients) [9]. In any case, these figures reflect the fact that in localized PDAC the classic molecular subtype prevails. In advanced PDAC, the proportion of tumors expressing GATA6 decreases, although this varies depending on the series, with figures ranging between 46% and 80% [9,10,11]. On the other hand, the expression of GATA4 has been less studied in PDAC. In our series, 42% of the tumors expressed GATA4, which is a lower percentage than that reported by other authors that place it between 68-71% [17,18]. This may be due to differences in patient characteristics and in the technique used.

Although initially it was considered that GATA6 could have an oncogenic role in PDAC because its amplification and over-expression contributed to cell proliferation and cell cycle progression [19,20], more recently, a tumor-suppressive role in PDAC mouse models has been proposed [21]. Furthermore, in human-derived organoids, GATA6 was found to be responsible for maintaining the classic transcriptional subtype, since its loss of expression is a robust surrogate biomarker for basal-like tumors [8,18,22,23]. Moreover, GATA6 expression reverted the basal-like subtype to the classical subtype, including the reversal of WNT-independent growth, a pathway that favors dissemination and metastasis [22]. Therefore, GATA6 exerts a pro-epithelial and anti-EMT function [8].

The role of GATA4 in PDAC development is also not well defined. It has been reported that GATA4 is aberrantly expressed in PDAC and that its over-expression could contribute to PDAC carcinogenesis by activating the Hedgehog and Notch pathways [17]. However, other authors have described that GATA4 inhibits the proliferation of PDAC tumor cells and favors cell differentiation [24]. It has recently been reported that, in the basal-like subtype, the expression of GATA4 is downregulated and that the low expression of GATA4 amplifies the transcriptomic effect of GATA6 loss [25].

Although GATA4 and GATA6 are involved in PDAC carcinogenesis [17], the results of our study suggest that their expression during carcinogenesis follows different pathways. When we compared the differences in expression between healthy tissue and PanIN, we found that GATA4 expression was decreased in a high percentage (44%) of cases, while GATA6 expression didn’t change. In this sense, it should be noted that in vitro studies suggest that GATA4 acts as a negative regulator in PDAC carcinogenesis and, therefore, its suppression may favor this process [24]. In addition, it has been pointed out that the deletion of GATA4 gives rise to an upregulation of the expression of GATA6, which would support the fact that the expression of GATA6 is maintained in most of the PanINs [15]. In addition, it has also been pointed out that in PanIN 2–3, an amplification of GATA6 is produced that promotes carcinogenesis and that it is maintained in the already established PDAC [18], which is in agreement with our observations, since in our series we did not detect differences in the expression of GATA4 or GATA6 between PanIN and tumor tissue [9]. However, when we investigated the differences between tumor tissue and lymph node metastasis, we found that while GATA4 expression remained stable in 71% of the cases, loss of GATA6 expression was common, being detected in up to 40% of cases. These data suggest that loss of GATA6 expression favors tumor invasiveness and spread. Indeed, in vivo studies indicate that when GATA6 expression is downregulated, the GATA6-regulated differentiation program is silenced, allowing transition to the basal-like/squamous subtype [26,27]. Also, GATA6 deletion in mice has been reported to dramatically increase the metastatic rate [28]. Furthermore, it should be noted that the loss of simultaneous expression of GATA4 and GATA6 has been reported to be related to the development of liver metastases [25].

In our study we have observed that the expression of these transcription factors was related to some clinical and pathological characteristics of the tumor. On the one hand, we observed a statistically significant association between patients with tumors that did not express GATA4 and a history of tobacco consumption. To our knowledge this finding has not been previously described in the reviewed literature, so it would have to be confirmed in other series. However, it should be noted that it has been reported that in mice, nicotine promotes pancreatic carcinogenesis and tumor development via the down-regulation of GATA6 [29], so it cannot be ruled out that GATA4 suppression may also be involved in tobacco induced carcinogenesis.

It should also be noted that all tumors diagnosed as stage IA/IB expressed GATA6, as was the case with well-differentiated tumors. However, due to their small number, no statistically significant differences were reached. Other authors have previously pointed out the relationship between the expression of GATA6 and the degree of differentiation [25]. Likewise, we observed that GATA6-positive tumors show less tendency to perineural invasion and less elevation of Ca 19.9 levels. All these results suggest that GATA6+ tumors have less aggressive characteristics, which may contribute to their better prognosis. However, the results obtained for GATA4 expression are more difficult to interpret, since GATA4 expression was associated with some poor prognostic factors such as perineural invasion and increased INL, but also with a lower elevation of Ca 19.9 and higher DFS. Other authors have also reported a positive relationship of the GATA4 expression with poor differentiation, although not with stage [24]. Although the relationship between GATA4 expression and female gender has been reported, we did not confirm this in our series [17].

The prognostic value of GATA6 has been previously reported by other authors, both in metastatic and resected disease [8,10,18,25]. In resected disease, patients with low GATA6 expression have been reported to have significantly lower OS than patients with moderate or high expression (4.6 vs. 12.7 vs. 13.1 months, respectively) [8]. In addition, the prognostic value of GATA6 was modified depending on the adjuvant treatment received after surgery, so that patients with tumors with low expression of GATA6 treated with regimens that included 5FU had a significantly worse prognosis than the rest. In contrast, survival in patients treated with adjuvant gemcitabine was independent of tumor expression of GATA6 [8]. These observations are consistent with the results of our study, where all patients received adjuvant gemcitabine and we were unable to detect differences in OS. However, when we analyzed DFS, we observed that it was related to both GATA6 and GATA4 expression. In fact, patients co-expressing GATA4/GATA6 had significantly higher DFS than those expressing only GATA6 or GATA4, and this, in turn, was significantly better than that of those who did not express GATA6 or GATA4. The fact that these DFS differences have no impact on OS is probably due to the fact that, after relapse, most patients received treatment with a 5FU-based regimen. In this regard, it should be noted that various studies suggest that the classic subtype is more sensitive to 5FU-based regimens than the basal-like subtype [5,10]. Since our study is the first to report data on the relationship between GATA4/6 expression and DFS, we cannot compare our results with those of other authors. It should be noted that in metastatic disease treated with gemcitabine regimens, differences in OS based on GATA6 expression could not be demonstrated either [11].

The main limitations of this study are the procedure used to determine the expression of GATA4 and GATA6 and the small sample size. Although it has been reported that GATA6 mRNA levels have been shown to accurately correlate with protein levels in human PDAC [10,11], the determination of the expression of transcription factors by IHC is not exempt from subjectivity. To reduce it, in our study, all the samples were evaluated independently by two expert pathologists, but, even so, a certain degree of variability cannot be ruled out. It has been suggested that the reliability of the determination can be improved by the use of a computer-assisted diagnosis program. Likewise, it is possible that the use of full sections instead of TMA cores reduces the variability produced by cellular heterogeneity. Therefore, given the small sample size of some subgroups, some of the associations observed in our work should be verified in larger series.

## 5. Conclusions

The results of our study suggest that determining the expression of GATA4 and GATA6 by IHC is feasible and provides clinical and prognostic information that can help improve patient stratification.

## Figures and Tables

**Table 1:** Baseline patients’ characteristics (*n* = 89).

Characteristics	Patients, n (%)
Age (Median, range)	64 (29–84)
Sex	
Male	43 (48)
Female	46 (52)
Comorbidities	
Diabetes	20 (23)
Chronic pancreatitis	32 (36)
Smoking	41 (46)
Alcohol use	28 (33)
TNM Stage	
IA	3 (3)
IB	5 (6)
IIA	15 (17)
IIB	66 (76)
Tumor grade	
Well differentiated	8 (9)
Moderately differentiated	72 (81)
Poorly differentiated	9 (10)
Lymph node invasion	66 (76)
Lymphovascular invasion	40 (45)
Perineural invasion	59 (66)
Surgical margins	
R0	39(44)
R1	50 (56)
Resected node nº (Median, SD)	16 (9.6)
Body mass index (Median, SD)	24 (3.9)
CA 19.9 UI/mL (Median, SD)	812 (1545)
INL	4.9 (14.8)
GATA6	
Positive	72 (81)
Negative	17 (19)
GATA4	
Positive	37 (42)
Negative	52 (58)

**Table 2:** Patients’ clinical characteristics and GATA6 & GATA4 expression.

Variable	GATA6			GATA4		
	Positive	Negative	*p*-Value	Positive	Negative	*p*-Value
Age (M, SD)	63 (12)	64 (14)	0.63	62 (12)	63 (12)	0.95
Sex						
Male	40	6	0.13	21	25	0.41
Female	32	11		16	27	
Diabetes						
Yes	16	5	0.53	8	12	0.87
No	56	12		29	40	
Chronic pancreatitis						
Yes	26	6	0.94	13	19	0.89
No	46	11		24	33	
Smoking						
Yes	31	10	0.24	11	30	**0.03**
No	41	7		26	22	
Alcohol use						
Yes	23	5	0.67	11	17	0.76
No	49	12		26	35	
TNM stage						
IA/IB	8	0	0.33	2	6	0.31
IIA/IIB	64	17		35	46	
Tumor grade						
G1	8	0	0.38	3	5	0.82
G2	57	15		30	42	
G3	7	2		4	5	
Lymph node invasion						
Yes	55	11	0.32	28	38	0.78
No	17	6		9	14	
Lymphovascular invasion						
Yes	32	8	0.84	18	22	0.55
No	40	9		19	30	
Perineural invasion						
Yes	44	15	0.065	34	25	**0.01**
No	28	2		3	27	
Surgical margins						
R0	29	7	0.94	16	20	0.65
R1	43	10		21	32	
CA 19.9 UI/mL (M, SD)	606 (1010)	1501 (2412)	**0.03**	284 (340)	982 (1610)	**0.01**
INL (M, SD)	5.3 (16.2)	3.24 (1.4)	0.36	7.8 (2.5)	3.5 (2.9)	**0.02**

Numbers in bold indicate significant *p*-values (*p* < 0.05).

**Table 3:** Univariate Cox proportional hazards regression survival analysis.

Variable	Disease Free Survival		Overall Survival	
	*p*	β (SE)	*p*	β (SE)
Age	0.535	−0.006 (0.010)	0.777	−0.003 (0.010)
Sex	0.896	0.034 (0.256)	0.819	−0.057 (0.248)
Diabetes	0.362	−0.259 (0.283)	0.205	−0.349 (0.276)
Chronic pancreatitis	0.707	−0.100 (0.267)	0.730	−0.091 (0.263)
Smoking	0.768	0.078 (0.265)	0.589	−0.140 (0.260)
Alcohol use	0.377	−0.136 (0.154)	0.990	0.002 (0.149)
Body mass index	0.404	−0.029 (0.035)	0.623	−0.017 (0.035)
TNM stage	**0.037**	0.433 (0.207)	0.589	0.244 (468)
Tumor grade	0.471	−0.203 (0.282)	0.152	−0.366 (0.255)
Lymph nodes invaded nº	**0.004**	0.134 (0.047)	0.120	0.073 (0.047)
Lymphovascular invasion	0.364	0.234 (0.258)	0.914	0.027 (0.248)
Perineural invasion	0.960	0.015 (0.287)	0.564	0.154 (0.266)
Surgical margins	0.793	0.068 (0.260)	0.623	0.124 (0.252)
CA 19.9	0.006	0.035 (0.012)	0.000	0.035 (0.009)
NLR	0.962	0.001 (0.022)	0.655	−0.009 (0.20)
GATA4 expression	**0.046**	0.652 (0.330)	0.902	0.043 (0.346)
GATA6 expression	**0.036**	0.254 (0.277)	0.366	0.239 (0.265)
GATA4/GATA6 expression	**0.018**	0.984 (0.298)	0.116	0.371 (0.295)

Numbers in bold indicate significant *p*-values (*p* < 0.05).

**Table 4:** Variables significantly associated with disease free survival in the Cox regression analysis.

Variable	β	SE	*p* †	HR (95% CI)
TNM stage	0.593	0.242	0.014	1.810 (1.127 to 2.908)
CA 19.9, g/dL	0.037	0.015	0.012	1.038 (1.006 to 1.268)
GATA4/6 expression	−0.952	0.315	0.031	0.386 (0.208 to 0.717)

† *p* values were calculated using a two-sided Wald test for multivariable analyses. CI = confidence interval; HR = hazard ratio.

## Data Availability

Anonymous individual data may be made available following publication by reasonable request to the corresponding author.
